# Feeding a High Concentration Diet Induces Unhealthy Alterations in the Composition and Metabolism of Ruminal Microbiota and Host Response in a Goat Model

**DOI:** 10.3389/fmicb.2017.00138

**Published:** 2017-02-02

**Authors:** Canfeng Hua, Jing Tian, Ping Tian, Rihua Cong, Yanwen Luo, Yali Geng, Shiyu Tao, Yingdong Ni, Ruqian Zhao

**Affiliations:** ^1^Key Laboratory of Animal Physiology and Biochemistry, Ministry of Agriculture, Nanjing Agricultural UniversityNanjing, China; ^2^College of Veterinary Medicine, Northwest A&F UniversityXianyang, China

**Keywords:** bacteria, gene expression/regulation, metabolism, goat, rumen

## Abstract

There is limited knowledge about the impact of long-term feeding a high-concentrate (HC) diet on rumen microbiota, metabolome, and host cell functions. In this study, a combination of mass spectrometry-based metabolomics techniques, 454 pyrosequencing of 16S rDNA genes, and RT-PCR was applied to evaluate the changes of ruminal microbiota composition, ruminal metabolites, and related genes expression in rumen epithelial cells of lactating goats received either a 35% concentrate diet or a 65% concentrate diet for 4 or 19 weeks, respectively. Results show that feeding a HC diet reduced the microbiota diversity and led to the disorders of metabolism in the rumen. The concentrations of lactate, phosphorus, NH3-N and endotoxin Lipopolysaccharide in ruminal fluids, and plasma histamine, lactate and urine N (UN) were increased significantly in goats fed with a HC diet. A significant increase of genes expression related to volatile fatty acids transport, cell apoptosis, and inflammatory responses were also observed in goats fed with a HC diet. Correlation analysis revealed some potential relationships between bacteria abundance and metabolites concentrations. Our findings indicate that a HC diet can induce ruminal microbiota dysbiosis and metabolic disorders, thus increasing risks to host health and potential harm to the environment.

## Introduction

A vast ensemble of ruminal microbiota including bacteria, archaea, ciliate protozoa, and anaerobic fungi provide important metabolic capabilities to digest cellulose-rich feedstuffs and to convert them into a wide range of nutrient compounds in order to sustain body maintenance and performance. A high-fiber diet and a stable microbiota community are necessary for keeping ruminants health. However, in the current feeding systems, particularly in the intensive management systems, it is a common strategy to feed large amounts of grains diet to ruminants due to the lack of quality forage and pursuing high milk yield (Bal et al., 1997; Soriano et al., 2000; Boerman et al., 2015). It is well known that feeding excessive amounts of non-structural carbohydrates and highly fermentable forages to ruminants commonly result in subacute ruminal acidosis (SARA), a common metabolic disease prevalent in high-producing animals (Klevenhusen et al., 2013). Previous evidence showed that SARA can be experimentally induced by feeding 50∼65% proportion of grain diet to ruminants (Tao et al., 2014a,b).

Animals suffering from SARA are usually accompanied with ruminal microbiota dysbiosis in bacteria, protozoa, anaerobic rumen fungi, archaea, and bacteriophages (Jami et al., 2013; Pitta et al., 2014). Currently, utilization of next generation sequencing technology (NGS) can describe the microbiome diversity and various factors that influence bacterial dynamics in greater resolution than ever before (Hristov et al., 2012). In previous studies (Mao et al., 2016), the alterations of ruminal microbiota were found in SARA ruminants fed with high-concentrate (HC) diets for a relatively short-term showing a decrease of Bacteroidetes, but an increase in Firmicutes. However, the effects of long-term feeding HC diets on the composition and structure of ruminal microbiota, metabolism and host responses are unknown. In this study, we used 454 pyrosequencing methods to investigate the changes of the structure and component of rumen microbiota in lactating dairy goats fed with a HC diet for short (4 weeks) or long (19 weeks) periods.

As one of the “core microbiome” (Mao et al., 2016), Firmicutes has a strong association with the biological fiber degradation (Koike et al., 2010). Methane is mainly produced by ruminal archaea, which is responsible for methane production in the rumen (Johnson and Johnson, 1995), and *Methanobrevibacter* is the most common genus in archaea (Poulsen et al., 2013). Methane is currently regarded as one of the most prevalent greenhouse gas, largely emitted from farm animal activities particularly from ruminants. Moreover, several toxic and inflammatory compounds were also found in the rumen (Saleem et al., 2012). Lipopolysaccharide (LPS) is typically released by the Gram-negative bacteria following the bacteria dying in the rumen (Mao et al., 2016). Histamine can alter rumen epithelial barrier and increases passive permeability (Mikelis et al., 2015).

Volatile fatty acids (VFAs) and microbial crude protein (MCP) are the principal products of bacteria fermentation (Russell et al., 1992). VFAs provide 70% of energy for ruminants, whereas excessive amount of VFAs will cause a considerable drop in rumen pH, push the activation of monocarboxylate transporters (MCTs), and other transport genes in the ruminal epithelium (Gabel et al., 2002). Mao et al. (2016) reported a significant decrease of saturated fatty acid and an increase of amine and phenylacetate concentrations in ruminal fluids of SARA animals. Rumen metabolic disorders associated with altering ruminal microbiota compositions are at high risks for developing diseases, particularly metabolic diseases including lameness, mastitis and laminitis (Zebeli and Metzler-Zebeli, 2012). Metabolomics can help us comprehensively understand the metabolism of microorganisms in the rumen.

Lipopolysaccharide may activate host cells via toll-like receptor 4 (TLR4) signaling pathway and induce the production and release of pro-inflammatory cytokines (Gruys et al., 2005). Feeding a HC diet for a short-term can induce the ruminal disturbance, a local inflammatory response in the ruminal epithelium, and even the systemic inflammation (Hook et al., 2011). However, information regarding the effects of long-term feeding HC diet on ruminal bacteria composition, metabolism, and the response of the epithelial cells has not been reported in ruminants. In this study, we used a combination of the 454 bar-coded pyrosequencing strategy and the gas chromatograph mass spectrometer (GC-MS) technique to investigate the effects of feeding a HC diet for short- and long-term on the alterations in ruminal microbiota and their metabolites, using goats as a model. Moreover, the relationships between microbiota abundance, ruminal metabolites, and genes expression related to host cells function were also analyzed in the present study.

## Materials and Methods

### Ethics

The Institutional Animal Care and Use Committee (IACUC) of Nanjing Agricultural University approved all animal procedures. The “Guidelines on Ethical Treatment of Experimental Animals” (2006) No. 398 set by the Ministry of Science and Technology, China and the Regulation regarding the Management and Treatment of Experimental Animals” (2008) No. 45 set by the Jiangsu Provincial People’s Government, was be strictly followed during the slaughter and sampling procedures.

### Animals and Experimental Procedures

In brief, 15 healthy, mid-lactating goats (Guanzhong dairy goats) with an average initial body weight of 49.7 ± 5.5 kg (mean ± SD) were housed in individual pens in a standard animal feeding house at Northwest A&F University (Shanxi, China). Prior to the experiment, all goats were allowed free access to a control diet containing a forage to concentrate ratio of 65:35 for 2 weeks. Ingredients and chemical composition of the experimental diets were shown in Supplementary Table S1. After dietary adaptation, goats were randomly assigned to two groups, goats in the control group (*n* = 10) fed with a low concentrate (LC) diet containing 65% forage and 35% mixed concentrate for 19 weeks. High-grain long-term group (HL) five goats received a high-grain diet containing 65% mixed concentrate and 35% forage for 19 weeks. After 13 weeks, five goats from LC control group were randomly assigned to the high-grain short-term (HS) group received the same diet as in HL group for 6 weeks including 2 weeks dietary adaptation. All goats were fed daily at 08:00 and 18:00, respectively.

### Samples Collection and Assay

At the end of the experiment, goats were slaughtered after overnight fasting. All goats were killed with neck vein injections of xylazine [0.5 mg (kg body weight)^-1^; Xylosol; Ogris Pharma, Wels, Austria] and pentobarbital [50 mg (kg body weight)^-1^; Release; WDT, Garbsen, Germany]. Blood samples were taken using heparin-containing vacuum tubes from jugular vein. Blood was centrifuged at 1,000 ×*g* for 15 min at 4°C, extracted plasma in EP and stored at -20°C. Immediately after slaughter, the abdominal cavity was opened by midline incision, after that the rumen was carefully removed. The rumen was opened from the dorsal side and rumen fluid was collected and strained through four layers of cheesecloth and kept on ice until processing. The rumen tissue was then washed by PBS, collected and threw into liquid nitrogen immediately, then stored at -80°C until next process.

The rumen fluid samples were briefly centrifuged at 10,000 ×*g* for 45 min at 4°C and the supernatant was aspirated gently to prevent its mixing with the pellet and passed through a disposable 0.22 μm LPS-free filter. The filtrate was collected in a sterile glass tube (previously heated at 180°C for 4 h) and heated at 100°C for 30 min. The ruminal LPS was detected by Chromogenic End-point Tachypleus Amebocyte Lysate Assay Kit (Chinese Horseshoe Crab Reagent Manufactory Co. Ltd, Xiamen, China) strictly following the manufacturer’s instructions. Histamine was detected with the enzyme linked immunosorbent assay kit (Shengxing Company, Nanjing, China) and strictly following the manufacturer’s instructions. The second portion of each rumen fluid sample was centrifuged at 3,000 ×*g* for 15 min at 4°C immediately after collection and the supernatant was collected to analyze VFAs concentrations. The level of MCP, UN, NH3-N, lactate, and ruminal capase-3 enzyme activity was detected with the commercial assay kits (Jiancheng Company, Nanjing, China) strictly following the manufacturer’s instructions, respectively.

### RNA Isolation, cDNA Synthesis and Real-Time PCR

Total RNA was extracted from rumen samples with Trizol Reagent (Takara, Dalian, China). Concentration and quality of total RNA were monitored by NanoDrop ND-1000 Spectrophotometer (Thermo, USA). Then, total RNA was treated with RNase-Free DNase (M6101, Promega, USA) and reverse transcribed. Two microliter of diluted cDNA (1:40, vol/vol) was used for real-time PCR, which was performed in Mx3000P (Stratagene, USA). GAPDH was not affected by the experimental factors, and was chosen as the reference gene. All primers used in this study were synthesized by Generay Company (Shanghai, China). The method of 2^-ΔΔCt^ was used to analyze the real-time PCR results and gene mRNA levels were expressed as the fold change relative to the mean value of control group. Primers sequences are listed in Supplementary Table S3.

### Metabolite Profiling of the Ruminal Fluid

The rumen fluid was thawed at room temperature, and centrifuged at 3,000 ×*g* for 15 min at 4°C. Two hundred microliter of the supernatant was removed to 1.5 mL centrifuge tube, mixed with 10 μL dichlorobenzene alanine, then centrifuged at 13,000 ×*g* for 15 min at 4°C, the supernatant removed to 200 μL tube. Twenty milligram methoxyammonium hydrochloride dissolved into 1 mL of pyridine was added to the sample after drying in 30 μL, and vortexed until completely dissolved, placed in 37°C incubator 90 min, then added 30 μL BSTFA at 70°C oven for 1 h.

Using Agilent7890A / 5975C GC-MS analysis of GC-MS platform for metabolomics sample data acquisition. Capillary column is the Agilent J&W Scientific’s HP-5 ms (30 m × 0.25 mm × 0.25 μm). Instrument parameters set as follows: inlet temperature of 280° C, EI ion source temperature of 230°C, quadrupole temperature 150°C, high purity helium (purity greater than 99.999%) as a carrier gas, splitless into Injection volume 1.0 μL. Temperature program: initial temperature of 80°C, maintaining the 2 min, 10°C/min speed was raised to 320°C, and maintained 6 min. Full scan mode using mass spectrometry, mass spectrometry in the range of 50–550 (m/z). Random sequence of consecutive samples analyzed, to avoid the impact due to signal fluctuations caused by the instrument.

XCMS package of R software was used to treat LC/MS data, and then the EXCEL2007 software was used to delete the impurity peaks. Finally, two-dimension data matrix data was obtained. The matrix was created through the SIMCA-P software (Version 13.0) to analyze PCA, PLS-DA and the loading plot in the end.

### DNA Extraction and 16S rDNA Gene Amplicon Pyrosequencing

Two-milliliter ruminal fluid was used for DNA extraction by PowerFecal^®^ DNA Isolation Kit (Mobio) strictly according to the manufacturer’s instructions. DNA samples were stored at -80°C for further processing. DNA purity was verified through agar gel electrophoresis, and was diluted to the concentration of 1.0 ng/μL. DNA was used as template to amplify the 16S V3-V4 region using specific primers with Barcode. The efficient hi-fi PCR enzyme and the Phusion^®^ High-Fidelity PCR Master Mix with GC Buffer (New England Biolabs) were added to insure the amplification efficiency and accuracy. The production of PCR was verified by electrophoresis in 1.5% agarose gel, and then recycled by gel extraction kit (qiagen). The database was made with TruSeq^®^ DNA PCR-Free Sample preparation Kit, and then used HiSeq2500 PE250 to sequencing.

Accordance to the Barcode and PCR primers sequences, raw tags were got by the FLASH (V1.2.7^1^. Further, high quality clean tags were obtained through strict filtering processing by the Qiime (V1.7.0^2^). The UCHIME Algorithm^3^ and gold database^4^ were used to detect chimeric sequences, removed them and acquired the effective tags. Effective tags were clustered to the Operational Taxonomic Units (OTUs) by Uparse (v7.0.1001^5^). The most abundant sequence within each OTU was designated as the representative sequence. The RDP classifier (Version2.2^6^) and GreenGene database^7^ were used to species annotation. PyNAST (Version 1.2) and Green Gene “Core Set” data information in the database were used for multiple sequence alignment to get the representative sequences’ phylogenetic relationship. Qiime (Version 1.7.0) was used to calculate the alpha diversity and the beta diversity. The unweighted PCoA analysis was carried out by the R (Version 2.15.3) with the WGCNA, stats and ggplot2 packages. V3–V4 region can detect the bacteria and archaea (Berg et al., 2012; Michelsen et al., 2014). We analyzed the bacteria and archaea, respectively.

### Statistical Analysis

Data are presented as means ± SEM. The data were tested for normal distribution and analyzed by Student’s unpaired *t*-test using SPSS software packages (SPSS version 19.0 for Windows; SPSS Inc., Chicago, IL, USA). Data were considered statistically significant when *P* < 0.05. The numbers of replicates used for statistics were noted in the figures. The correlation was made by the corrplot package of the R software.

## Results and Discussion

### Alteration of VFAs and Abnormal Fermented Products after Feeding a HC Diet

Feeding a HC diet to lactating goats induced abnormal fermentation in rumen. Although there were no significant alternations of VFA concentrations in ruminal fluid among LC, HS, and HL groups, most of them showed a decrease in HC fed goats compared to LC. The ratio of acetate/propionate (A/P) was also decreased in HC goats and reached a significant decrease in HL group (*P* = 0.04) compared to LC (**Table 1**), indicating the alternation of fermentation type in rumen. Ruminal NH3-N, UN, phosphorus, and LPS concentrations were significantly increased in HS and HL goats (*P* < 0.05; **Table 1**). Compared to LC, ruminal lactate (LA) and MCP, as well as plasma histamine, LA, and UN concentrations also increased in animals fed with HC diet, particularly in the HL group (*P* < 0.05; **Table 1**).

**Table 1:** Concentrations of metabolites in ruminal fluid and plasma.

Measure	LC	HS	HL
**In ruminal fluid**
Acetate (mM)	8.2352 ± 0.7522	5.8716 ± 0.9132	5.7813 ± 1.1244
Propionate (mM)	3.5036 ± 0.2896	2.8031 ± 0.5507	2.8426 ± 0.5261
Isobutyrate (mM)	0.3511 ± 0.0330	0.3227 ± 0.0445	0.3341 ± 0.0304
Butyrate (mM)	2.3261 ± 0.0658	2.400 ± 0.5426	2.3593 ± 0.4137
Isovalerate (mM)	0.4291 ± 0.0445	0.3901 ± 0.0601	0.4142 ± 0.0398
Valerate (mM)	0.2360 ± 0.0124	0.2480 ± 0.0348	0.2702 ± 0.0473
Total VFA (mM)	15.1951 ± 1.0374	12.1353 ± 2.0902	12.1216 ± 2.1649
Acetate/Propionate	2.3595 ± 0.1251^a^	2.1647 ± 0.1444^ab^	2.0338 ± 0.0740^b^
Lactate (mM)	0.6684 ± 0.1276^b^	0.8394 ± 0.1074^ab^	1.0925 ± 0.0818^a^
NH3-N (mM)	9.6200 ± 1.2525^b^	16.9000 ± 1.5161^a^	19.1743 ± 1.2010^a^
MCP (mg/mL)	5.1521 ± 0.0880^b^	5.8364 ± 0.3110^ab^	5.7729 ± 0.1168^a^
UN (mM)	56.8734 ± 8.1717^b^	93.5949 ± 6.0290^a^	99.8101 ± 7.7480^a^
Phosphorus (mM)	3.5474 ± 0.5678^b^	5.9605 ± 0.0750^a^	6.0517 ± 0.0948^a^
Histamine (ng/mL)	122.3936 ± 8.7446	98.4652 ± 12.6695	112.1426 ± 9.9324
LPS (EU/mL)	95663.3081 ± 10939.1852^b^	130604.5340 ± 5279.7075^a^	127962.8463 ± 4932.3793^a^
**In plasma**
Histamine (ng/mL)	185.5518 ± 32.6975^b^	280.8482 ± 29.6947^ab^	309.8263 ± 27.3023^a^
Phosphorus (mM)	0.2375 ± 0.0097	0.3099 ± 0.0846	0.2675 ± 0.0710
Lactate (mM)	6.7154 ± 0.2644^b^	7.7365 ± 0.3434^a^	7.5701 ± 0.6118^ab^
UN (mM)	8.7595 ± 0.4860^b^	9.9114 ± 0.8405^ab^	10.3888 ± 0.4660^a^
Urine UN(mM)	56.038 ± 5.4666	52.7089 ± 6.6299	53.7342 ± 4.3704

*Values are expressed as the means ± SEM. Value with different small letter superscripts mean significant difference compared to LC (*P* < 0.05), and with the same or no letter superscripts mean no significant difference (*P* > 0.05). VFA, volatile fatty acids; NH3-N, Ammonia-nitrogen; MCP, microbial crude protein; UN, urine nitrogen; LPS, lipopolysaccharide.*

Although VFAs concentrations in ruminal fluids were generally decreased in HC fed goats compared to LC goats, the total amount of VFA was not markedly affected by the HC diet, which was consistent with the earlier studies (Sun et al., 2015). It was reported that a lower proportion of acetate to propionate was caused by a lower fermentation of cellulose in rumen (Ribeiro et al., 2005). In this study, a significant decrease of the ratio of acetate to propionate in HL goats may indicate a lower cellulose fermentation in rumen. Moreover, increasing the proportion of butyrate in the current study was consistent with previous studies *in vitro* (Vallimont et al., 2004; Ribeiro et al., 2005) and *in vivo* (Martel et al., 2011; Oba et al., 2015). Unaffected production of branched-chain VFA (isobutyrate and isovalerate) was consistent with earlier studies (Li et al., 2014). It is widely accepted that large amount of lactate leads to an acute ruminal acidosis, and lactate is one of major products in a rapid fermentation process (Nagaraja and Titgemeyer, 2007). Lactate can cross the rumen wall and be dissolved in blood to leading an increase of plasma lactate as observed in the HS and the HL groups.

Absorption and utilization of nitrogen is promoted in lactating goat by feeding a HC diet. Our results demonstrated that the concentration of NH3-N was remarkably increased in the HS and HL groups compared to the LC (**Table 1**), which was consistent with the previous studies (DeFrain et al., 2004; Ariko et al., 2015). Increased nitrogen suggests a more intense proteolysis (Bunthof et al., 2001). We did not find significant changes of UN in urine among the control, the HS, and the HL groups, indicating that urea might be utilized in other metabolic pathways. We found that ruminal phosphate concentration was significantly increased in the HS and the HL groups, compared to the control group (**Table 1**). However, feeding a HC diet did not change the level of phosphate in blood. Therefore, we speculated that excessive amount of ruminal phosphate was eliminated from the body through urine or feces, which will throw a potential risk to environment through methane emission.

It’s well known that some metabolic diseases occurred in ruminants including acidotic rumenitis (Underwood, 1992) and laminitis (Nilsson, 1963), have been found to directly correlate with the level of endogenous histamine. In the Groot’s (1998) study, histamine altered rumen epithelial barrier function by trans-epithelial electrolyte transportation and increased the passive permeability. In this study, the level of histamine in plasma was markedly increased in the HS and HL groups compared to control. High level of endogenous histamine and endogenous LPS pose a high risk to induce metabolic related diseases in lactating ruminants after digested a HC diet, as observed in the farm practice.

### Alteration of Ruminal Flora

**FIGURE 1:**
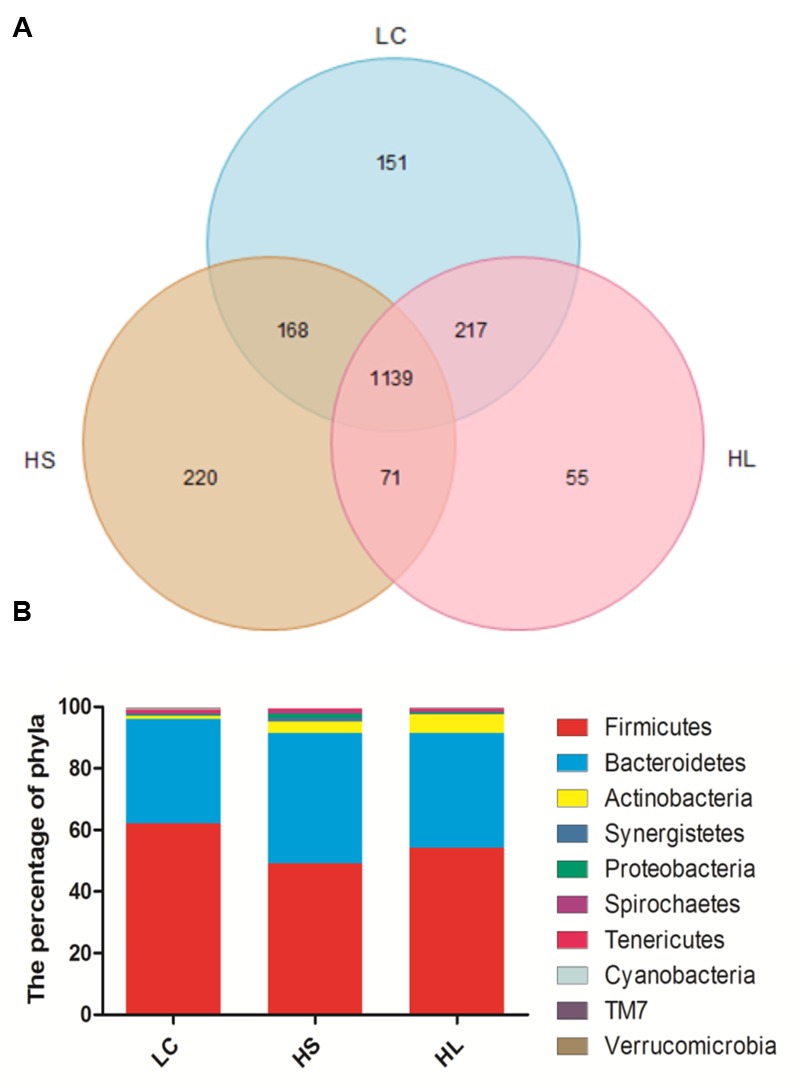
**(A)** Venn diagram demonstrates overlap of bacterial OTUs at 3% dissimilarity level for low concentrate (LC), high-grain short-term (HS) and High-grain long-term group (HL) group. **(B)** Effects of length of concentrate to forage diets on the compositions of microbial phylum (as a percentage of the total sequence).

In total, 910,696 reads were obtained for the bacterial 16S rRNA genes by pyrosequencing analysis. After screening these gene sequences with strict criteria, 702,001 valid sequences were obtained, accounting for 86.62% of the raw reads. The common shared numbers among three groups were shown in Venn diagram (**Figure 1A**). The HS group had the highest number of unique sequences (220 OTUs), followed by the LC group (151 OTUs) and the HL group (55 OTUs). Additionally, there were 1139 OTUs (around 56% of total OTUs) shared among three groups. At phylum level, Firmicutes was the most abundant bacteria, with an average relative abundance of 55.10% (**Figure 1B**). Bacteroidetes were the second type with an average relative abundance of 37.82% (**Figure 1B**). We observed a notable phylum-wide shift in the Cyanobacteria and Verrucomicrobia induced by the HC diet. The Cyanobacteria abundance was significantly decreased in the HS group compared to the LC group, while Verrucomicrobia was significantly decreased in the HL group compared to the HS (**Figure 1B**).

**FIGURE 2:**
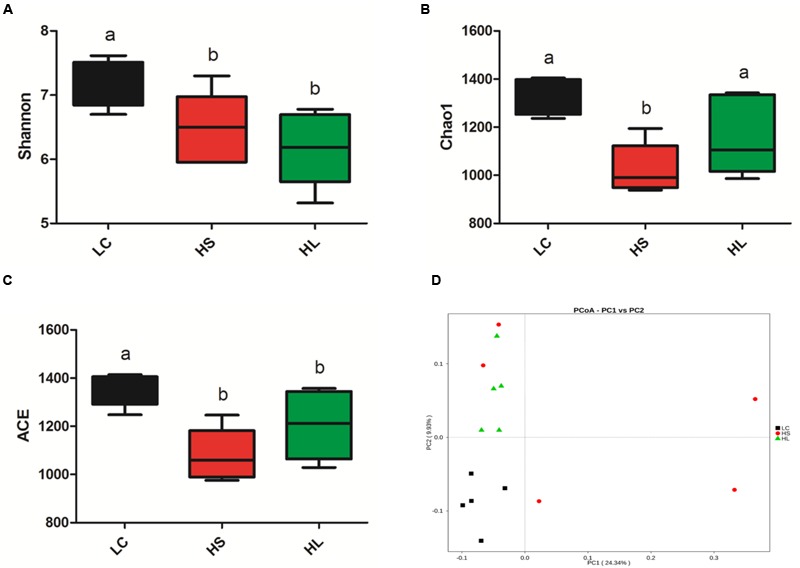
**Changes in ruminal microbial richness and diversity as a result of LC, HS and HL. (A)** The bacterial richness estimated by the Shannon index. **(B)** The bacterial diversity estimated by the Chao1. **(C)** The bacterial diversity estimated by the ACE. **(D)** Unweighted principal coordinate analysis (PCOA) of rumen bacteria microbiota.

The diversity of bacteria in rumen fluid was present by Shannon index, Chao value, and ACE index. Shannon index was markedly reduced in HS (*P* = 0.02) and HL (*P* < 0.01) groups compared to LC control group (**Figure 2A**). Chao1 values were observed in the HS group (*P* < 0.01; **Figure 2B**). ACE values were significantly decreased in the HS (*P* < 0.01) and HL (*P* = 0.02) groups (**Figure 2C**). Taken above together, feeding a HC diet induces a dramatic decrease of the diversity of ruminal bacteria particularly in long-term feeding group.

Previous studies demonstrated that feeding a HC diet for 4∼6 weeks altered ruminal microbiota, which commonly caused a negative impact on the bio-diverse ecosystem (Mao et al., 2016). We found that PCoA (**Figure 2D**) can notably show the significant differences of ruminal bacterial composition among control, HS, and HL groups, which demonstrated that feeding a diet shifted from 35% concentrate to 65% proportion for 4 weeks changed the bacterial composition abruptly and unstably. However, after 19 weeks adaptation microbiota compositions maintained stability, but exhibited a lower richness and diversity than LC control group. The PCoA with unweighted UniFrac distances demonstrated that samples from LC group were clearly separated from HS and the HL groups. Moreover, the intragroup variation in HS goats was obviously presented by PCoA analysis indicating an unstable composition of ruminal microbiota.

Ruminants fed with HC diets are associated with an altered rumen microbiota. **Supplementary Figure S1** shows that at genus level, there is 10 genus markedly changed among top 50 abundance OTUs by HC diet (*P* < 0.10). The abundance of *Bulleidia, Paracoccus, Pseudoramibacter*_*Eubacterium, Atopobium*, and *Selenomonas* were increased through the HC diets (**Supplementary Figure S1**). However, the level of *Oscillospira, YRC22, Bacteroides, CF231*, and *Akkermansia* were significantly decreased in the HC group (**Supplementary Figure S1**). In Mao et al. (2016) study, the population of *Prevotella, Papillibacter, Lysinibacillus, Thalassospira, Succiniclasticum*, as well as some unclassified bacteria were decreased in the HC groups, while the abundance of *Butyrivibrio, Mogibacterium, Acetitomaculum*, and unclassified *Anaerolineaceae* were increased by the HC diet. The changes in the percentage of bacterial are approximately 20∼40% in Mao et al. (2016) study. The alterations of the bacteria were approximately 4%. The alteration of ruminal flora caused by the origin of animals and different management (Petri et al., 2013). High abundance of OTUs alteration could replace other OTUs that take over the analogous functions (Taxis et al., 2015), and geographical differences could also possibly affect the composition of bacteria.

The abundance of *Selenomonas, Atopobium, Bulleidia, Paracoccus*, and *Pseudoramibacter_Eubacterium* were increased. *Selenomonas* is classified into two subspecies (subsp): subsp. *lactilytica* with a capacity to utilize lactate, and incapable of utilizing lactate subsp. *ruminantium* (Asanuma et al., 2015). However, *Selenomonas ruminantium* is a representative nitrate and nitrite reducing ruminal bacterium (Asanuma et al., 2002). It is reported that an increase in nitrate and nitrite in ruminal contents induced the abundance increase of *Selenomonas ruminantium* (Asanuma et al., 2015). The increase of lactate and “N” nutrients can contribute to the increase of *Selenomonas.* As a Gram-positive anaerobic bacterium, *Atopobium* utilizes sugars and plays an important role in developing SARA (Harmsen et al., 2000; Mao et al., 2013). In this study, we found that the population of *Atopobium* was increased by feeding the HC diet, as reported in previous studies (Mao et al., 2013). It is consistently found that feeding a HC diet is likely to induce SARA disorders and other related metabolic diseases in ruminants, particularly in lactating dairy cows (Mao et al., 2016).

We found that the level of *Bacteroides, Oscillospira, Akkermansia, CF231*, and *YRC22* were decreased by the HC diet. *Bacteroides* is one of ureolytic bacteria in rumen (Slyter et al., 1968) and can produce succinic acid (Davies et al., 2007). Zhou et al. (2012) reported that the reduction potential can inhibit the growth of *Bacteroides*. In the present study, UN was considerably increased, possibly contributing to the decrease of *Bacteroides*. As a Gram-positive bacterium, *Oscillospira* is the first described bacterium (Chatton and Pérard, 1913) involved in the degradation of plant cell wall (Yanagita et al., 2003). Mackie et al. (2003) found that the abundance of *Oscillospira* in rumen was diet-dependent and reaching the maximum level after feeding fresh-forage diets. *Akkermansia* is involved in mucosa development, as well as maintenance of intestinal integrity by utilizing mucin and antimicrobial (Everard et al., 2013). Moreover, the decrease of *CF231* and *YRC22* in ruminal fluids may be caused by higher level of nitrate (Zhao et al., 2015).

**FIGURE 3:**
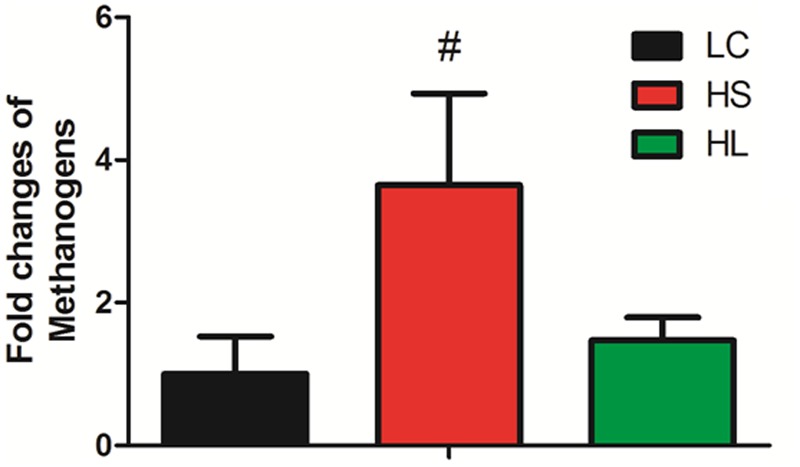
**Methanogens change.** Methanogens change in three groups. Value with # superscripts means *P* < 0.1 compared to LC, and without # superscript means *P* > 0.1.

It’s very important to note that the abundance of methanogens has a trend to increase in the HS group compared to LC (**Figure 3**). Mao et al. (2016) reported that SARA ruminants produced a higher level of methanogens than control healthy counterparts. Inconsistently, in Hook et al. (2011) study four non-lactating Holstein dairy cows fed with a HC diet for 3 weeks did not affect the methanogen density in the rumen. We found that the level of methanogens exhibited an increased tendency in the HS group. As the prevalent greenhouse gas, methane is highly produced by ruminants and has a potentially harmful effect on the environment.

### Shift of Metabolomics in Ruminal Fluid

**FIGURE 4:**
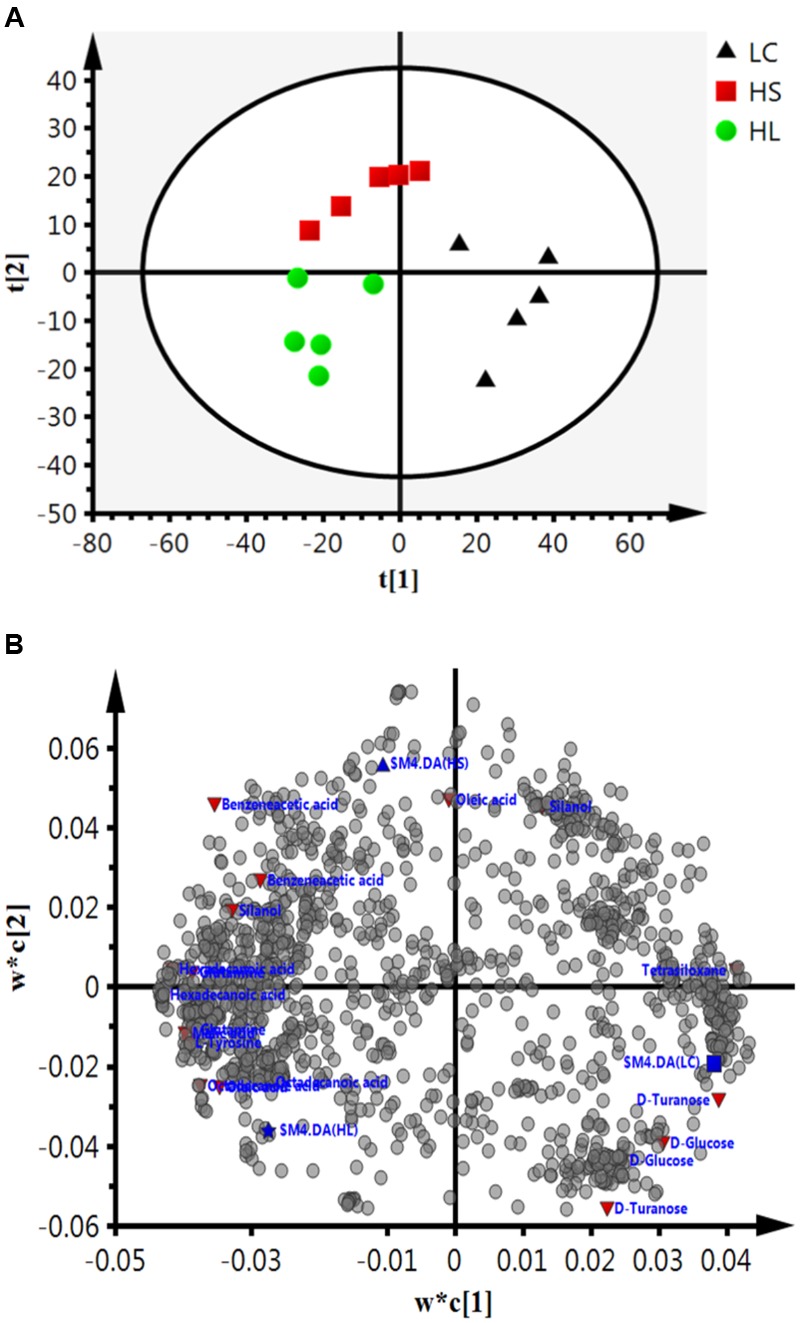
**Partial least squares discriminant analysis (PLS-DA) based on the rumen compounds data. (A)** PLS-DA analysis scores plots discriminating among the rumen fluid LC, HS and HL. The different color represents different group. **(B)** Loading plot of all the commonly detected compounds projected into the PLS-DA model. The most important compounds responsible for the discrimination are labeled and colored in red.

Gas chromatograph mass spectrometer chromatograms of the ruminal fluid metabolites were displayed in **Supplementary Figure S2**. Numbers of visible peaks were separated by GC-MS analysis. After pairwise comparison, 31, 30, and 16 differentiated metabolites with VIP > 1 between two groups was presented in Supplementary Table S2. These metabolites altered by HC diet are involved in multiple biochemical processes in the rumen, such as gluconeogenesis. PLS-DA was used to identify the key compounds responsible for the score differences. There was an obvious separation between the score plots created by the first two components and three groups of samples cluster (**Figure 4A**). Each dot represents an observation sample, and the distance between two dots represents the similarity of the sample’s metabolite composition. We can observe the LC group cluster presented in the right portion; HS and HL groups were presented in the upper and lower portion of the left, respectively.

In order to verify the differentiated metabolites among LC, HS, and HL groups, a PLS-DA loading plot was created (**Figure 4B**). The variable importance in the projection (VIP) combining with the student’s *t*-test (*t*-test) *p* values were used to identify which compounds was the most significant contribution in discriminating among the ruminal compounds of groups. Those compounds that were responsible (VIP > 1) for the significant difference between LC-HS, LC-HL and HS-HL were selected. Finally, 11 compounds were commonly differentiated among three groups (**Table 2**), and three of them including D-glucose, D-turanose and tetrasiloxane were significantly decreased, whereas eight metabolites including benzeneacetic acid, glutamine, hexadecanoic acid, L-tyrosine, malic acid, octadecanoic acid, oleic acid, and silanol were increased by feeding the HC diet. Silanol and tetrasiloxane may be derived from the forage, because they do not exist in any metabolic pathways. In this study, the relative density of phenylacetate acid in rumen was higher in the HS and the HL groups, which was consistent with the previous findings (Mao et al., 2016). In rumen, phenylacetate was synthesized by activity of ruminal microbiota by degrading plant constituents (Chesson et al., 1999). It has been reported that phenylacetate acid could be beneficial to the rumen bacteria (Kristensen, 1974). Our findings reveal a correlated response for phenylacetate and the proportion of some ruminal bacteria, strongly positive correlated with *Oscillospira* and *Akkermansia*, but negatively correlated with *Paracoccus*. The above results indicate that these microbes may be affected by phenylacetate, or collaboratively synthesize phenylacetate.

**Table 2:** Candidate ruminal compounds that significantly different among three groups.

Item	LC vs. HS			LC vs. HL		
	VIP	*P*	Fold change	VIP	*P*	Fold change
Phenylacetate acid	1.2442	0.0114	0.9493	1.1084	0.0155	0.7775
Hexadecanoic acid	1.5586	0.0000	0.3944	1.3527	0.0004	0.4954
Octadecanoic acid	1.1061	0.0332	0.4656	1.0265	0.0304	1.1477
Oleic acid	1.4849	0.0003	0.8938	1.2445	0.0033	1.7250
Glutamine	1.2610	0.0097	0.4141	1.3523	0.0004	0.5153
L-tyrosine	1.2843	0.0077	0.8296	1.3728	0.0003	1.2852
Malic acid	1.2673	0.0091	0.7655	1.3785	0.0002	1.1701
D-Glucose	1.3008	0.0065	-0.8998	1.0012	0.0364	-0.1499
D-Turanose	1.5266	0.0001	-1.1963	1.2900	0.0016	-0.9936

*VIP, variable importance in the projection.*

Feeding a HC diet to dairy goats dramatically reduced the level of D-glucose and D-turanose, which are both utilized by microbes in rumen (Evans and Martin, 2000). After absorption, propionate is utilized for synthesis glucose in liver. More utilization and less synthesis finally led to a decrease of D-glucose. D-Turanose can be metabolized by the bacteria as well, such as *Elusimicrobium minutum* (Geissinger et al., 2009) and *Bacillus* (Seo et al., 2013). Two amino acids, glutamine and L-tyrosine were identified in ruminal fluids. The greater concentration of glutamine in the HL group suggested more proteins degraded after feeding a HC diet. Both glutamine and L-tyrosine exhibited strong correlations with microbes including *Bulleidia, Oscillospira, YRC22, CF231*, and *Akkermansia.* It’s reasonable to speculate that these microbes may play an important role in producing or utilizing these two amino acids. In the current study, the amount of malic acid in the rumen was dramatically increased in the HS and HL groups compared to the control group, and a greater concentration of oleic acid and octadecanoic acid was also found in HC-fed goats. Malic acid can promote the utilization of lactate by *Selenomonas ruminantium* and then can prevent the decrease of ruminal pH (Castillo et al., 2004). Moreover, malic acid can increase the production of propionate (Khampa et al., 2006) and butyrate (Liu et al., 2009), and regulate the activity of some types of ruminal bacteria (Liu et al., 2009). The increase of octadecanoic acid comes from the hydrogenation of oleic acid, and the abundance of *Bulleidia, Oscillospira, CF231* and *Paracoccus* is strongly correlated with the level of hexadecanoic acid and oleic acid.

### Changes of Functional Genes Expression in Host Cells

**FIGURE 5:**
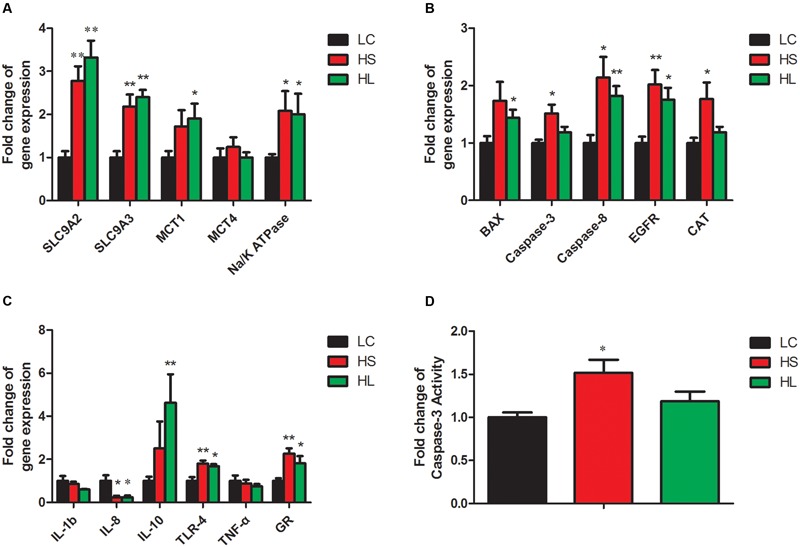
**Gene expressions in rumen.** GAPDH was used as the reference gene for the gene expressions. **(A)** Genes involved in substrates transport. **(B)** Genes involved in apoptosis, proliferation and antioxidant process. **(C)** Genes involved in inflammation response. Value with different small letter superscripts mean significant difference (*P* < 0.05), and with the same or no letter superscripts mean no significant difference (*P* > 0.05). **(D)** Effects of length of concentration to forage diets on concentration of caspase-3 activity.

In the present study, the results showed that the functional genes expressions in host epithelial tissues changed greatly by feeding a HC diet. The expression of VFAs transport genes including sodium/hydrogen exchanger 2 (*SLC9A2*), sodium/hydrogen exchanger 3 (*SLC9A3)*, and sodium-potassium adenosine triphosphatase (*Na/K ATPase*) in ruminal epithelium was significantly increased by feeding HC diet (**Figure 5A**). It’s well known that intracellular pH is regulated in a certain physiological range, which is very important within cell homeostasis (Counillon and Pouysségur, 2000). Kiela et al. (2001) has reported that epidermal growth factor receptor (*EGFR*) can promote SLC9A2 expression. Consistently, we also found a significant increase of EGFR mRNA expression in ruminal epithelial tissues in HC-fed goats (**Figure 5B**). Monocarboxylate transporter 1 (*MCT1*) and 4 (*MCT4*) are involved in the transmembrane transport (Kirat et al., 2006; Connor et al., 2010). Taken together, the altered genes expression in the epithelial tissues indicate the changes of physiological functions including immunity, substrates transportation, as well as the homeostasis of the cross-talk between ruminal microbiota and the host proceeding the HC diet to lactating dairy goats.

**FIGURE 6:**
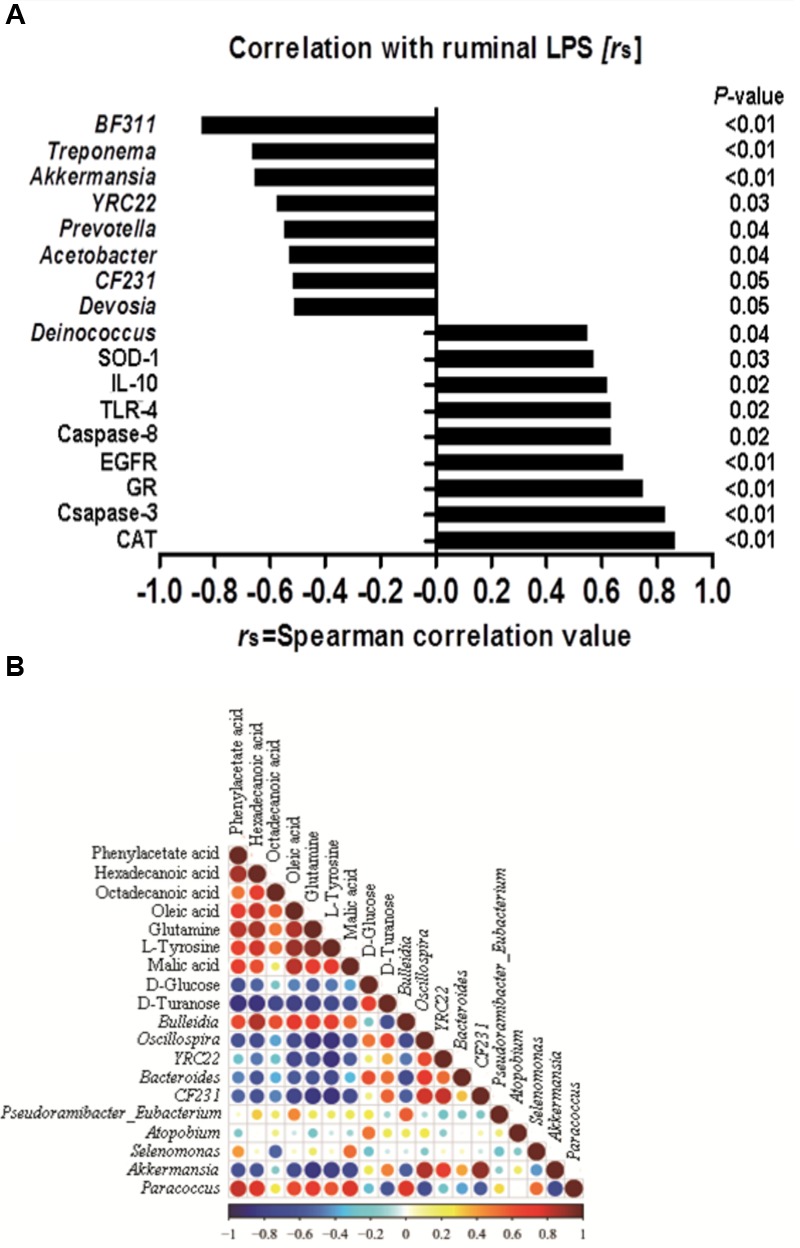
**(A)** Correlation among the ruminal Lipopolysaccharide (LPS), bacteria, genes and the potential marker compounds. **(B)** Correlation analysis of 100 most abundant OTUs and the genes with LPS, only the significant (*P* < 0.05) correlations are depicted.

Caspase-8 and the downstream effector caspase-3 (Strater et al., 1997), as well as B-cell lymphoma (*Bcl-2*) family (Nagata, 1997) were involved in the apoptotic process. In the present study, caspase-8 and *BCL-2* associated X (*BAX*) mRNA expression were significantly increased in the HS and the HL groups compared to LC group (**Figure 5B**). Caspase-3 mRNA and enzyme activity (**Figure 5D**) were not significantly changed in the HL group, however, we found a strong positive correlation of caspase-3 and -8 with ruminal LPS levels. As an antioxidant enzyme, catalase (CAT) gene expression in epithelial tissues was significantly increased in the HS goats (**Figure 5B**), and showed a strong positive correlation with ruminal LPS level (**Figure 6A**). Inflammation is a major host defense reaction. The cytokines play a key role in the initiation, maintenance, and termination of the inflammatory reaction. Tumor necrosis factor-α (*TNF-α*), interleukin-1β (*IL-1β*), interleukin-6 (*IL-6*) and 8 (*IL-8*) are pro-inflammatory, while interleukin-10 (*IL-10*) is anti-inflammatory (Wojdasiewicz et al., 2014). The expression of IL-8 mRNA was markedly decreased, whereas the expression of IL-10 and glucocorticoid (GR) gene was dramatically increased in the host epithelial tissues after feeding a HC diet (**Figure 5C**). It’s well known that LPS stimulates the expression of pro-inflammatory cytokines via TLR4 signal pathway (Lu et al., 2008). However, most of cytokines expression were not altered in the HL group, which probably indicates the high tolerance of the host to ruminal endotoxin gradually, and eventually attenuate LPS responses (Ametaj et al., 2009).

### Correlation between Ruminal LPS, Genes Expression and Microbiome

A correlation analysis of the 100 most abundant OTUs and genes expression with ruminal LPS levels is shown in **Figure 6A**. LPS is a structural component of the cell wall of Gram-negative bacteria, recognized by TLR-4, and activates the immune response (Gruys et al., 2005). The increase of LPS always accompanies the reduction of Gram-negative bacteria (Khafipour et al., 2009). In this study, there are nine OTUs among the 100 most abundant OTUs, which is substantially correlated with LPS level (*p* < 0.05). All of these nine OTUs are Gram-negative bacteria. Moreover, our results showed that the level of ruminal LPS also depicted a strong correlation with epithelial apoptosis, proliferation, inflammation, and anti-oxidative stress in the host rumen tissues (*r* > 0.6, *p* < 0.05).

### Correlation between Ruminal Microbiome and Metabolome

Correlation analysis within or between the bacteria and metabolite was conducted to investigate the potential co-occurrences as displayed with a correlation matrix (**Figure 6B**). In this context, 66 correlations (34.73%) were strong positively or negatively correlated (|*r*|≥ 0.6, *p* < 0.05). There were 31 correlations strong positively or negatively correlated (|*r*|≥ 0.6, *p* < 0.05) between OTUs and metabolite. Hexadecanoic acid and *Bulleidia* had the strongest positive correlation (*r* = 0.84, *p* < 0.01), and the strongest negative correlation were found between L-tyrosine and *Oscillospira* (*r* = -0.879, *p* < 0.01) in the bacteria and metabolite.

## Conclusion

Feeding a HC diet to lactating ruminants induces abnormal fermentation, metabolic perturbations, and microbiota dysbiosis with reduced bacterial richness and diversity. The microbiota dysbiosis in the HS group is more severe than that in the HL group presented by PCoA analysis. Microbiota dysbiosis and the abnormal products, particularly LPS and histamine, in rumen or circulating system induced by a HC diet is largely associated with functional genes expression in ruminal epithelial tissues. Therefore, the homeostasis of ruminal microbial ecosystem is a vital point for keeping host cells physiological functions, animal welfare, and even the healthy environment.

## Author Contributions

Performed experiments: CH, YG, JT, PT, ST, YL, and RC. Analyzed data: CH, JT, and ST. Conceived and designed experiments: YG, YN, RZ, RC, PT, and JT. Wrote the paper: CH and JT.

## Conflict of Interest Statement

The authors declare that the research was conducted in the absence of any commercial or financial relationships that could be construed as a potential conflict of interest.

## Footnotes

1 http://ccb.jhu.edu/software/FLASH/

2 http://qiime.org/scripts/split_libraries_fastq.html

3 http://www.drive5.com/usearch/manual/uchime_algo.html

4 http://drive5.com/uchime/uchime_download.html

5 http://drive5.com/uparse/

6 http://sourceforge.net/projects/rdp-classifier/

7 http://greengenes.lbl.gov/cgi-bin/nph-index.cgi

## Supplementary Material

FIGURE S1 **The shift in the percentage of bacteria taxa in rumen bacteria community (*P* < 0.10)**.

FIGURE S2 **Total ion chromatograms of ruminal fluid GC-MS corresponding with LC, HS, and HL groups**.
