# Supplementary figures and images for: Feeding a High Concentration Diet Induces Unhealthy Alterations in the Composition and Metabolism of Ruminal Microbiota and Host Response in a Goat Model
Source: Front Microbiol. 2017 Feb 2;8:138. doi: 10.3389/fmicb.2017.00138 (PMC5288341; doi:10.3389/fmicb.2017.00138)

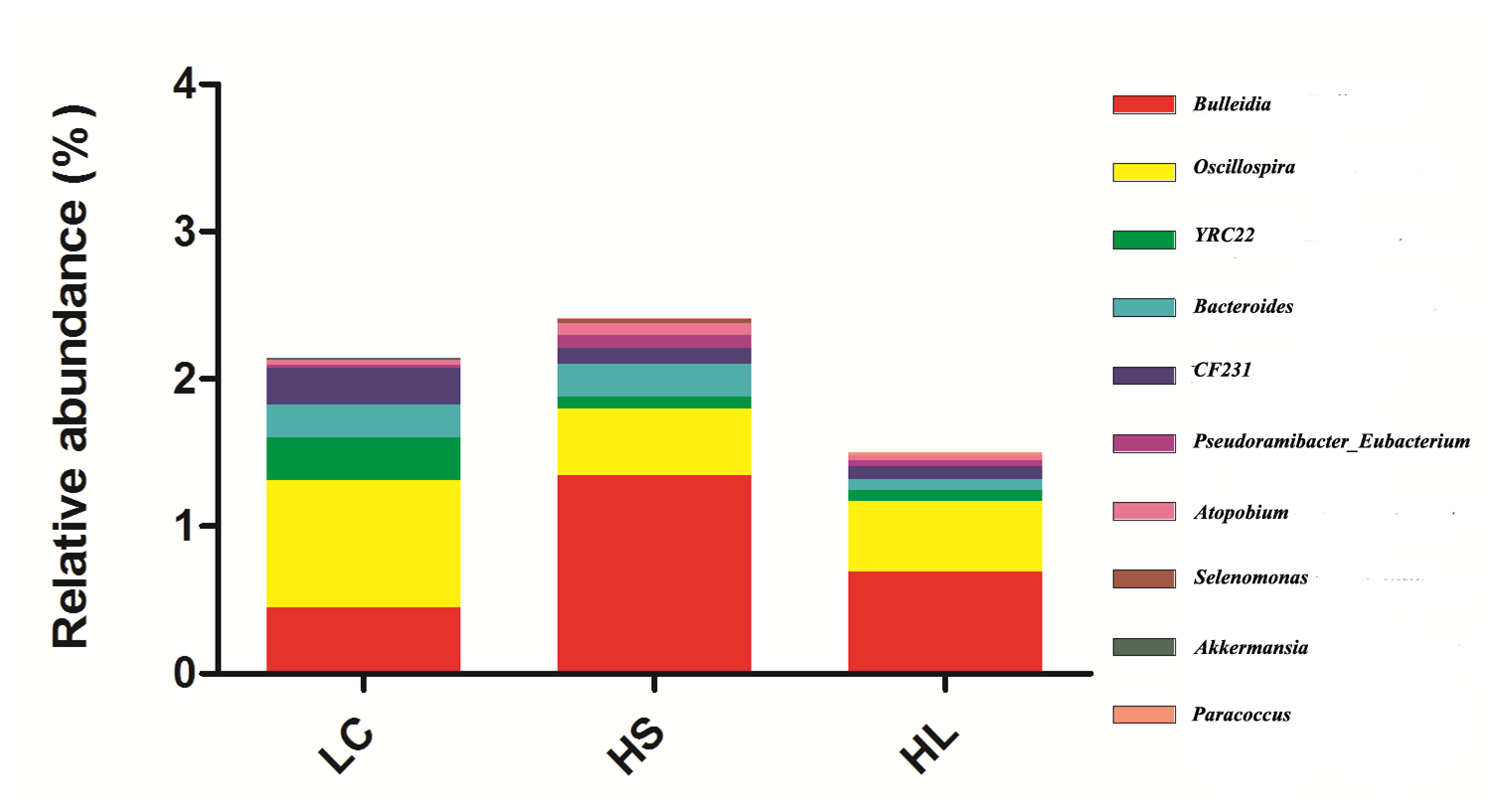

Supplement: FIGURE S1 — The shift in the percentage of bacteria taxa in rumen bacteria community (P < 0.10). [file Image_1.TIF]

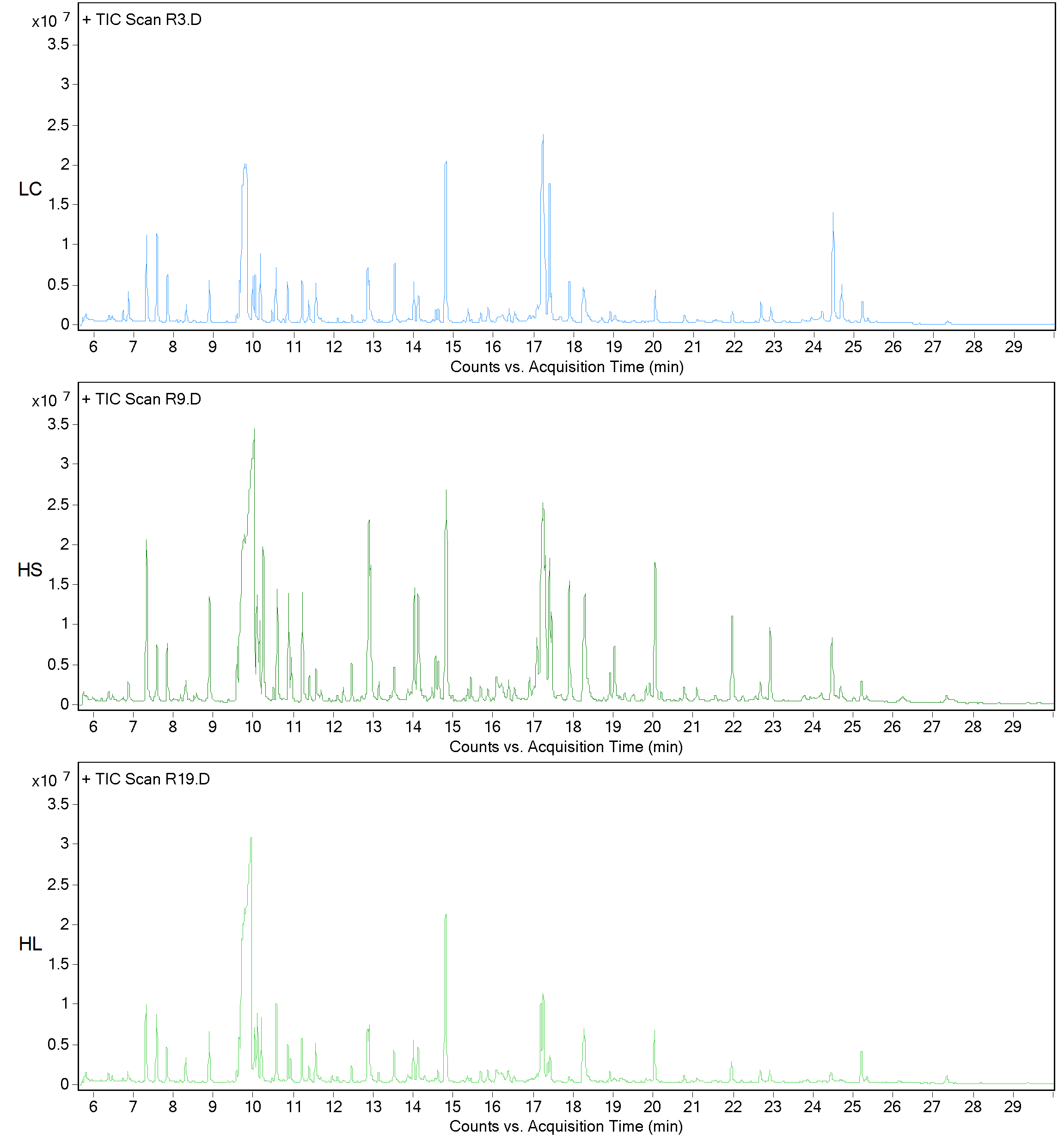

Supplement: FIGURE S2 — Total ion chromatograms of ruminal fluid GC-MS corresponding with LC, HS, and HL groups. [file Image_2.TIF]
