# Supplementary material for: Feeding a High Concentration Diet Induces Unhealthy Alterations in the Composition and Metabolism of Ruminal Microbiota and Host Response in a Goat Model
Source: Front Microbiol. 2017 Feb 2;8:138. doi: 10.3389/fmicb.2017.00138 (PMC5288341; doi:10.3389/fmicb.2017.00138)
Supplement: Supplementary file 3 [file Table_1.DOCX]

| **Table S1** Ingredients and composition of the experimental diets (%) | | | | |
| --- | --- | --- | --- | --- |
| **Items** |  | **The ratio of concentrate to forage** | | |
|  |  | **35:65** |  | **65:35** |
| Ingredients (% of DM) |  |  |  |  |
| Corn silage |  | 43.00 |  | 23.00 |
| Medicago sativa hay |  | 22.00 |  | 12.00 |
| Corn |  | 22.98 |  | 22.00 |
| Wheat bran |  | 0.00 |  | 33.90 |
| Soybean meal |  | 9.72 |  | 6.50 |
| Limestone meal |  | 0.40 |  | 1.40 |
| Calcium phosphate dibasic |  | 1.00 |  | 0.30 |
| Salt |  | 0.40 |  | 0.40 |
| Premix |  | 0.50 |  | 0.50 |
| Total |  | 100 |  | 100 |
| Nutrient levels^2^ (%) |  |  |  |  |
| Net energy(MJ/kg) |  | 5.75 |  | 5.87 |
| Digestible crude protein |  | 8.22 |  | 8.2 |
| Crude protein |  | 13.70 |  | 13.70 |
| Neutral detergent fiber |  | 43.06 |  | 38.67 |
| Acid detergent fiber |  | 26.31 |  | 19.88 |
| Calcium |  | 0.87 |  | 0.96 |
| Phosphorus |  | 0.43 |  | 0.49 |

1Provided per kg of premix: Vitamin A 6 000U; Vitamin D2 500U; Vitamin E 80 mg; Cu 6.25 mg; Fe 62.5 mg; Zn 62.5 mg; Mn 50 mg; I 0.125 mg; Co 0.125 mg; Mo 0.125 mg.

^2^ Nutrient levels were estimated from the current goat foods.
