# Supplementary material for: Feeding a High Concentration Diet Induces Unhealthy Alterations in the Composition and Metabolism of Ruminal Microbiota and Host Response in a Goat Model
Source: Front Microbiol. 2017 Feb 2;8:138. doi: 10.3389/fmicb.2017.00138 (PMC5288341; doi:10.3389/fmicb.2017.00138)
Supplement: Supplementary file 4 [file Table_2.DOCX]

Table S2. The alterations in relative density of ruminal fluid metabolites in goats correspond with LC-HS, LC-HL and HS-HL.

| LC-HS |  |  |  |
| --- | --- | --- | --- |
|  | VIP | P | Fold changes |
| Amino acid |  |  |  |
| Glutamine | 1.2610 | 0.0097 | 0.4141 |
| L-Aspartic acid | 1.1606 | 0.0227 | 0.5703 |
| L-Tyrosine | 1.2843 | 0.0077 | 0.8296 |
| L-Valine | 1.2845 | 0.0077 | 0.5335 |
| L-Phenylalanine | 1.1742 | 0.0204 | 0.4475 |
| L-Leucine | 1.1831 | 0.0191 | 0.5065 |
| Organic acid |  |  |  |
| Malic acid | 1.2673 | 0.0091 | 0.7655 |
| Hexanoic acid | 1.4752 | 0.0004 | -0.7410 |
| Benzenepropanoic acid | 1.1694 | 0.0212 | -0.4644 |
| Hexadecanoic acid | 1.5586 | 0.0000 | 0.3945 |
| Octadecanoic acid | 1.1061 | 0.0332 | 0.4656 |
| 3-Pyridinecarboxylic acid | 1.3805 | 0.0024 | -0.4005 |
| Benzoic acid | 1.3637 | 0.0030 | -1.8122 |
| Azelaic acid | 1.3585 | 0.0033 | -0.5725 |
| Oleic acid | 1.4849 | 0.0004 | 0.8938 |
| Benzeneacetic acid | 1.2442 | 0.0114 | 0.9493 |
| Pentanoic acid | 1.1196 | 0.0303 | -0.4535 |
| Silicide |  |  |  |
| Silanamine | 1.1042 | 0.0336 | 0.8612 |
| Silanol | 1.1759 | 0.0202 | 0.2209 |
| Octasilsesquioxane | 1.2358 | 0.0123 | 0.5026 |
| Silane | 1.4470 | 0.0008 | -0.8495 |
| Pentasiloxane | 1.0839 | 0.0383 | 0.5868 |
| Tetrasiloxane | 1.4382 | 0.0009 | -0.4595 |
| Sugar |  |  |  |
| D-Glucose | 1.3008 | 0.0065 | -0.8998 |
| D-Turanose | 1.5266 | 0.0001 | -1.1963 |
| D-Galactose | 1.1678 | 0.0215 | -0.8652 |
| Mineral acid |  |  |  |
| Phosphoric acid | 1.4048 | 0.0017 | 1.4868 |
| Sugar alcohol |  |  |  |
| Myo-Inositol | 1.0507 | 0.0469 | 0.8767 |
| Nucleosides and nucleotides |  |  |  |
| 9H-Purine | 1.3808 | 0.0024 | 0.3464 |
| Adenosine | 1.2783 | 0.0082 | 2.3143 |
|  |  |  |  |
|  |  |  |  |
|  |  |  |  |
| LC-HL |  |  |  |
|  | VIP | P | Fold changes |
| Amino acid |  |  |  |
| Glutamine | 1.3523 | 0.0004 | 0.5153 |
| L-Aspartic acid | 1.0351 | 0.0285 | 0.3208 |
| L-Tyrosine | 1.3728 | 0.0003 | 1.2852 |
| L-Valine | 1.3722 | 0.0003 | 0.6990 |
| L-Phenylalanine | 1.2760 | 0.0020 | 0.5426 |
| L-Leucine | 1.3302 | 0.0007 | 0.7051 |
| L-Threonine | 1.0866 | 0.0188 | 0.6481 |
| L-Isoleucine | 1.0899 | 0.0183 | -0.5967 |
| Serine | 1.3065 | 0.0012 | 0.7655 |
| Organic acid |  |  |  |
| Pantothenic acid | 1.0491 | 0.0256 | 0.5765 |
| Malic acid | 1.3785 | 0.0002 | 1.1701 |
| Hexanoic acid | 1.3396 | 0.0006 | -1.0908 |
| Benzenepropanoic acid | 1.2216 | 0.0045 | -0.5801 |
| Hexadecanoic acid | 1.3527 | 0.0004 | 0.4954 |
| Octadecanoic acid | 1.0265 | 0.0304 | 1.1477 |
| 3-Pyridinecarboxylic acid | 1.0973 | 0.0171 | -0.4606 |
| Benzoic acid | 1.3345 | 0.0007 | -2.9624 |
| Azelaic acid | 1.3158 | 0.0010 | -0.7397 |
| Oleic acid | 1.2445 | 0.0033 | 1.7250 |
| Benzeneacetic acid | 1.1084 | 0.0155 | 0.7775 |
| Pentanoic acid | 1.3936 | 0.0001 | -0.8803 |
| Silicide |  |  |  |
| Silanamine | 1.0728 | 0.0211 | 0.7996 |
| Silanol | 1.0697 | 0.0217 | 0.5469 |
| Silane | 1.3395 | 0.0006 | -0.9341 |
| Pentasiloxane | 1.3198 | 0.0009 | 0.8160 |
| Tetrasiloxane | 1.3677 | 0.0003 | -0.7770 |
| Sugar |  |  |  |
| D-Glucose | 1.0012 | 0.0364 | -0.1499 |
| D-Turanose | 1.2900 | 0.0016 | -0.9936 |
| Mineral acid |  |  |  |
| Phosphoric acid | 1.0542 | 0.0246 | 1.7121 |
| [Sugar alcohol](https://en.wikipedia.org/wiki/Sugar_alcohol) |  |  |  |
| Myo-Inositol | 1.2075 | 0.0054 | 1.1469 |
| Nucleosides and nucleotides |  |  |  |
| 9H-Purine | 1.0032 | 0.0359 | 0.2171 |
|  |  |  |  |
| HS-HL |  |  |  |
|  | VIP | P | Fold changes |
| Amino acid |  |  |  |
| Glutamine | 1.3071 | 0.0460 | 0.2868 |
| L-Tyrosine | 1.3790 | 0.0320 | 0.4556 |
| L-Threonine | 1.6890 | 0.0030 | 1.1360 |
| L-Isoleucine | 1.5189 | 0.0130 | -0.4798 |
| Serine | 1.7343 | 0.0020 | 0.6235 |
| Organic acid |  |  |  |
| Malic acid | 1.4074 | 0.0270 | 0.4046 |
| Hexadecanoic acid | 1.4418 | 0.0220 | 0.1729 |
| Octadecanoic acid | 1.5166 | 0.0140 | 0.6536 |
| Oleic acid | 1.3541 | 0.0360 | 0.8312 |
| Benzeneacetic acid | 1.4097 | 0.0270 | -0.4092 |
| Silicide |  |  |  |
| Octasilsesquioxane | 1.3371 | 0.0400 | -0.5522 |
| Silane | 1.6107 | 0.0070 | -0.3025 |
| Tetrasiloxane | 1.3390 | 0.0390 | -0.3176 |
| Sugar |  |  |  |
| D-Glucose | 1.3742 | 0.0330 | 0.5157 |
| D-Turanose | 1.4521 | 0.0208 | 1.4992 |
| Nucleosides and nucleotides |  |  |  |
| Adenosine | 1.6029 | 0.0070 | -2.4359 |
|  | | | |
