# Supplementary material for: Feeding a High Concentration Diet Induces Unhealthy Alterations in the Composition and Metabolism of Ruminal Microbiota and Host Response in a Goat Model
Source: Front Microbiol. 2017 Feb 2;8:138. doi: 10.3389/fmicb.2017.00138 (PMC5288341; doi:10.3389/fmicb.2017.00138)
Supplement: Supplementary file 5 [file Table_3.DOCX]

Table S3. Realtime-PCR primers used in this study

| Gene | Primer sequence (5’-3’)^a^ | Product length (bp) | Reference/GenBank accession |
| --- | --- | --- | --- |
| TLR4 | F: GTTTCCACAAGAGCCGTAA | 195 | ([Tao et al., 2014](#_ENREF_85)) |
|  | R: TGTTCAGAAGGCGATAGAGT |  |  |
| IL-1β | F: GAAGAGCTGCACCCAACA | 172 | ([Tao et al., 2014](#_ENREF_85)) |
|  | R: CAGGTCATCATCACGGAAG |  |  |
| TNF-α | F: CAAGTAACAAGCCGGTAGCCC | 173 | ([Tao et al., 2014](#_ENREF_85)) |
|  | R: CCTGAAGAGGACCTGCGAGTAG |  |  |
| IL-8 | F: GAACTTCGATGCCAATGC | 216 | JN 559767.1 |
|  | R: TCATGGATCTTGCTTCTCAG |  |  |
| IL-10 | F: CTATTTGGGAAGAAGCC | 218 | XM_005690416 |
|  | R: AGGGCAGTCAGGGAAAA |  |  |
| Caspase-3 | F: GGTTCATCCAGGCTCTTT | 98 | ([Tao et al., 2014](#_ENREF_85)) |
|  | R: TTCTGTCGCTACCTTTCG |  |  |
| Caspase-8 | F: GGCTCCTCTGAGATGCTG | 149 | ([Tao et al., 2014](#_ENREF_85)) |
|  | R: TGCTCCCGTGCTATGCTAT |  |  |
| Bax | F: TGCTCACTGCCTCACTCAC | 178 | ([Tao et al., 2014](#_ENREF_86)) |
|  | R: CCAAGACCACTCCTCCCCAT |  |  |
| NHE2 | F: GGTCATATTCTTCACTGTCTTC | 93 | XM_604493.9 |
|  | R: GCTTGCTGCTTCTTATTGG |  |  |
| NHE3 | F: AAGAACCTGTTTGTCAGCACCAC | 108 | NM_001192154.1 |
|  | R: TTCACTTCTCTTCACCTTCAGCC |  |  |
| MCT1 | F: CTTGGCAG ACCTTTATCCTC | 168 | ([Koho et al., 2011](#_ENREF_44)) |
|  | R: CTCCACAATGGTCACCAATCC |  |  |
| MCT4 | F: CTACAGAGCCTGAGAAGAACGG | 118 | NM_001109980.1 |
|  | R: GCTAAATACGAGCGTTGACGG |  |  |
| Na/K ATPase | F: CCTCGAAATCCATTGCTTATACC | 137 | NM_001076798.1 |
|  | R: GACCATGTCCGTTCCCAAGT |  |  |
| EGFR | F: TGAAAAACAGTGCAAGGCCG | 157 | ([Steele et al., 2015](#_ENREF_79)) |
|  | R: ACGACTGAAGTTCTGGCAGG |  |  |
| GR | F: GGAATAGATGCCAAGGGTC | 169 | NM_001114186 |
|  | R: CAGAGTTTGGGAGGTGGTC |  |  |
| GAPDH | F: GGGTCATCATCTCTGCACCT | 180 | ([Tao et al., 2014](#_ENREF_86)) |
|  | R: GGTCATAAGTCCCTCCACGA |  |  |

Koho, N. , Taponen, J. , Tiihonen, H. , Manninen, M., and Pösö, A. (2011) Effects of age and concentrate feeding on the expression of MCT 1 and CD147 in the gastrointestinal tract of goats and Hereford finishing beef bulls. *Res. Vet. Sci.* 90, 301-305. DOI: 10.1016/j.rvsc.2010.06.002

Steele, M. , Schiestel, C. , AlZahal, O. , Dionissopoulos, L. , Laarman, A. , Matthews, J., et al. (2015) The periparturient period is associated with structural and transcriptomic adaptations of rumen papillae in dairy cattle. *J. Dairy Sci.* 98, 2583-2595. DOI: 10.3168/jds.2014-8640

Tao, S. , Duanmu, Y. , Dong, H. , Ni, Y. , Chen, J. , Shen, X., et al. (2014) High concentrate diet induced mucosal injuries by enhancing epithelial apoptosis and inflammatory response in the hindgut of goats. *PLoS ONE.* 9. DOI: 10.1371/journal.pone.0111596

Tao, S. , Duanmu, Y. , Dong, H. , Tian, J. , Ni, Y., and Zhao, R. (2014) A high-concentrate diet induced colonic epithelial barrier disruption is associated with the activating of cell apoptosis in lactating goats. *BMC Vet. Res.* 10, 1. DOI: 10.1186/s12917-014-0235-2
